# Association between chronic disease and catastrophic health expenditure in Korea

**DOI:** 10.1186/s12913-014-0675-1

**Published:** 2015-01-22

**Authors:** Jae-Woo Choi, Jong-Won Choi, Jae-Hyun Kim, Ki-Bong Yoo, Eun-Cheol Park

**Affiliations:** Department of Public Health, College of Medicine, Yonsei University, Seoul, Korea; Institute of Health Services Research, College of Medicine, Yonsei University, Seoul, Korea; Department of Preventive Medicine, College of Medicine, Yonsei University, Seoul, Korea; Division of Business Administration, Yonsei University, Wonju, Korea; Department of Preventive Medicine and Institute of Health Services Research, Yonsei University College of Medicine, 50 Yonsei-ro, Seodaemun-gu Seoul, 120-752 Korea

## Abstract

**Background:**

The prevalence and economic burden of chronic diseases are increasing worldwide. Nevertheless, little information is available on catastrophic health expenditures (CHE) associated with chronic diseases in Korea. This study explored the burden of household out-of-pocket health expenditures among the Korean population for different chronic diseases.

**Methods:**

This study was conducted utilizing data collected from the 7,006 households that participated in the Korea Health Panel Survey (KHPS) in 2008. The effect of CHE in relation to type of chronic disease was assessed via multiple logistic regression analysis.

**Results:**

Roughly 3.5% of the participating households experienced CHE. As opposed to households headed by females and middle-aged individuals (40–59 years), those of low economic status, elderly households, and households with a member who suffered from a chronic disease were more likely to experience CHE. According to type of chronic condition, households with a member who suffered from cerebrovascular disease, diabetes, or chronic kidney disease were at a significantly higher risk of experiencing CHE.

**Conclusion:**

Although Korea has greatly expanded its health insurance coverage, financial protection against CHE remains a concern. Policy-makers need to focus on expanding benefits according to the economic burden of individual chronic conditions.

## Background

Korea achieved universal health insurance coverage just 12 years after its national health insurance system was implemented. However, most studies on the impact of health insurance in Korea have found that insurance has had only a modest effect on reducing out-of-pocket (OOP) payments [1-4]. The modest impact of insurance coverage on financial protection reflects the fact that people with health insurance are still paying quite high OOP payments. The proportion of total medical spending financed by the public sector is only 58%, which is lower than the OECD average (72%), and is the fourth lowest OECD level of spending after Chile (47%), Mexico (48%), and the USA (48%) [5].

When studying medical expenditure, in addition to considering the ratio of non-insured medical costs, it is important to examine OOP burden relative to other developed countries [6,7]. A high proportion of individual or family contributions typically indicate inequality issues in health and medical treatment systems, particularly in health finance systems. More importantly, a high OOP burden with respect to income could result in financial catastrophe for individuals or households [8]. High expenditures could lead to a reduction in spending on basic goods, such as food, while also decreasing money available for education [9].

Many households fall into poverty due to OOP medical spending [10-12]. Despite relatively low benefit coverage, by OECD standards, Korea is experiencing a sharp increase in medical expenditures due to an aging population and costs associated with utilizing new medical technologies [13]. This increase in medical costs often results in catastrophic medical fees that exceed the amount households can afford [3,4].

Catastrophic health expenditure is one index used to measure the burden of medical costs upon households at a national level; it estimates the proportion of households that spend a certain amount of income or consumption expenditure on health costs. A high incidence rate of catastrophic health expenditure reveals the inadequacies of medical social security systems in achieving their goal of protecting household finances [14]. Therefore, for Korea to attain its goal of medical social security, greater understanding of catastrophic health expenditure is needed. Accordingly, a number of studies on catastrophic health expenditure in Korea have been undertaken: previous studies have mainly provided evidence for the incidence of household catastrophic health expenditure [15], factors influencing the recurrence of household catastrophic health expenditure [16], analysis of private health insurance on catastrophic health expenditure among households with a cancer patient [17], impact of the benefit extension policy on catastrophic health care expenditure [18], relationship between catastrophic health expenditures and household income, and expenditure patterns in South Korea [19].

Meanwhile, few studies have investigated the association between chronic diseases and catastrophic health expenditure. Medical costs differ in terms of amount and health outcome, depending on the type of chronic disease present. Some diseases result in a high financial burden concentrated within a short time period, whereas other diseases result in costs that are both steady and high over the life of a patient. Studying the effect of different types of chronic diseases upon catastrophic health expenditure may aid in strengthening disease-dependent benefit coverage. Thus, this paper attempted to delineate the rate of catastrophic health expenditure, identify factors associated therewith, and characterize the burden of catastrophic health expenditure due to chronic disease status.

## Methods

### Sources of data

This study utilized data collected from the Korea Health Panel Survey (KHPS), which comprises nationally representative data and is publicly available (KIHSA and KNHIC 2009). The KHPS collected data for a total of about 8,000 households, with a response rate of 94.1%. The KHPS alleviated problems with recall bias by using household financial record books or receipts of health care spending. The KHPS data includes information on demographic and socio-economic characteristics of individuals, each event of health care utilization and expenditure, and details on chronic status, including the type of chronic disease. The current study was approved of by the Institutional Review Board (IRB) of Yonsei University Graduate School of Public Health.

### Definition of major variables

The main independent variable for this study was the type of chronic disease, and we included seven chronic diseases (neoplasm, cerebrovascular disease, ischemic heart disease, diabetes, arthritis, chronic kidney disease, and hypertension). These diseases were selected on the basis of their high medical costs [20]. The neoplasm variable included all cancers and reflected whether or not a neoplasm existed at the household level. In contrast, variables for the other chronic diseases reflected whether someone suffered from a condition at the individual level.

In this study, we used several covariates to control for demographic and socio-economic characteristics and health status. We included factors such as the sex, age, income, education level, economic activities, household composition, health insurance type, and the existence of chronic disease within a household.

The dependent variable was catastrophic health expenditure defined by the World Health Organization (WHO). According to the WHO, catastrophic health expenditure occurs when direct health care spending exceeds 40% of a household’s capacity to pay, and the WHO specifies that the standard should be altered appropriately for each country [21]. We defined a household’s capacity to pay as the amount of money spent per month, excluding food expenses, using the methods described in Xu et al. [10]. Meanwhile, catastrophic health expenditure, as derived by Xu et al. [10], is defined as health expenditure that is 40% greater than the ability of the household to pay [10]. When defining catastrophic health expenditure, the actual capacity to pay of a household must be measured accurately. Unfortunately, in the Korea Health Panel data, only income and living expenses are categorized, and thus, we were unable to calculate catastrophic health expenditure according to the method proposed by Xu et al. [10]. In this research, we overcame this limitation by calculating food expenses by referring to Korea National Statistics. We estimated Korea’s catastrophic health expenditure rate by incorporating estimated food expenses into our consideration of living expenses vs. medical expenditures for each household.

An OOP health payment is a payment made by a household when receiving a health service. Medical and drug costs resulting from emergency and outpatient care, as well as hospitalizations, were included as OOP payments in this study, while transportation and nursing costs were excluded.

### Statistical analysis

We analyzed the catastrophic health expenditure rate of Korean households according to their socioeconomic status and type of chronic disease. To identify factors associated with catastrophic health expenditure and, in particular, to examine the relationship between chronic diseases and catastrophic health expenditure, we conducted multiple logistic regression analysis. In addition, we analyzed the association between membership in Korea’s medical aid program, which is similar to Medicaid in the United State of America, and catastrophic health expenditure for households in the lowest 5, 10, 15, and 20% of incomes.

## Results

This research was carried out utilizing data collected via biannual surveys of 7,866 households, comprising 24,616 individuals. In this paper, we included a total of 7,006 households that responded to both surveys. Roughly 3.5% of households in the Korea Panel data experienced catastrophic health expenditures in 2008. This figure appears reliable and is similar to that reported by the National Statistical Office for the same year (3.0%). We found that catastrophic health expenditure was more frequent in households headed by women than men (5.6 vs. 3.2%). In addition, households with older or less educated heads had a higher rate of catastrophic health expenditure. The rate of catastrophic health expenditure was highest among households in the first economic quintile (i.e., those in the lowest income bracket). Households that contained only adults and those in which the head was insured through their workplace or regional plans experienced low levels of catastrophic health expenditure, while households with elderly members, uninsured members, or members suffering from chronic diseases had higher rates of catastrophic health expenditure (Table 1). To identify factors associated with catastrophic health expenditure, we conducted logistic regression analysis, using catastrophic health expenditure as a dependent variable and socioeconomic characteristics as independent variables. Households headed by females and middle-aged individuals (40–59 years) had significantly lower catastrophic health expenditure rates than those headed by males or younger/older individuals. In addition, households that benefited from Korea’s medical aid program had significantly lower catastrophic health expenditure rates. In contrast, households of low economic status, elderly households, and households containing members with chronic diseases were at significantly increased odds of experiencing catastrophic health expenditure (Table 2).

**Table 1:** **General characteristics and catastrophic health expenditure**

**Variables**	**Catastrophic health expenditure occurrence**		**Catastrophic health expenditure non-occurrence**		**Total**	**p-value**
	**By household**		**By household**			
	**Frequency**	**%**	**Frequency**	**%**		
Sex of head of household						<.0001
Male	188	3.2	5,752	96.8	5,940	
Female	60	5.6	1,006	94.4	1,066	
Age of head of household (years)						<.0001
20-39	6	0.4	1,458	99.6	1,464	
40-59	42	1.3	3,216	98.7	3,258	
60-79	183	8.5	1,980	91.5	2,163	
Economic level of household						<.0001
Third quintile	4	0.2	2,332	99.8	2,336	
Second quintile	17	0.7	2,392	99.3	2,409	
First quintile	227	10.0	2,034	90.0	2,261	
Education level of head of household						<.0001
Less than elementary school	123	8.1	1,399	91.9	1,522	
Middle school	41	4.9	803	95.1	844	
High school	53	2.2	2,357	97.8	2,410	
Greater than college	31	1.4	2,199	98.6	2,230	
Head of household’s job type						<.0001
Regular employee	44	1.7	2,587	98.3	2,631	
Temporary employee	46	3.6	1,234	96.4	1,280	
Employer or owner/operator	122	4.9	2,357	95.1	2,479	
Unpaid family worker	2	3.6	53	96.4	55	
Household composition						<.0001
Adult household (15–624 years old)	206	3.3	6,098	96.7	6,304	
Senior citizen who lives alone	24	15.4	131	84.6	155	
Elderly household	11	27.5	29	72.5	40	
Type of medical insurance						0.6761
Self-employed or employees	230	3.5	6,355	96.5	6,585	
Medical aid beneficiaries	14	4.1	323	95.9	337	
Uninsured, self-pay	4	4.8	80	95.2	84	
Chronic disease present in household?						<.0001
Yes	242	4.6	4,996	95.4	5,238	
No	6	0.3	1,761	99.7	1,767	
Total	248	3.5	6,758	96.5	7,006

**Table 2:** **Factors associated with catastrophic health expenditure occurrence**

**Variables**	**Unadjusted**		**Adjusted**	
	**OR**	**95% CI**	**OR**	**95% CI**
Sex of head of household				
Male	1.000		1.000	
Female	1.825	(1.354–2.459)	0.495	(0.340–0.721)
Age of head of household				
20–39	1.000		1.000	
40–59	0.286	(0.200–0.408)	0.667	(0.453–0.982)
60–79	1.989	(1.495–2.645)	1.062	(0.749–1.507)
Economic level of household				
Third quintile	1.000		1.000	
Second quintile	4.143	(1.392-12.332)	1.555	(1.540-1.570)
First quintile	65.064	(24.167-175.164)	16.375	(16.322-16.429)
Education level of head of household				
Greater than college	1.000		1.000	
Less than elementary school	6.237	(4.182–9.300)	0.808	(0.501–1.304)
Middle school	3.622	(2.256–5.815)	0.796	(0.470–1.348)
High school	1.595	(1.020–2.494)	0.734	(0.452–1.191)
Head of household’s job type				
Regular employee	1.000		1.000	
Temporary employee	1.488	(1.028–2.155)	0.842	(0.564–1.259))
Employer/owner operator	2.066	(1.548–2.759)	0.975	(0.705–1.348)
Unpaid family worker	1.507	(0.361–6.293)	0.552	(0.126–2.411)
Household composition				
Adult household (15–64 years old)	1.000		1.000	
Senior citizen who lives alone	5.677	(3.598–8.957)	1.720	(0.955–3.098)
Elderly household	11.750	(5.793–23.836)	2.775	(1.238–6.219)
Type of medical insurance				
Self-employed or employees	1.000		1.000	
Medical aid beneficiaries	1.198	(0.690–2.078)	0.283	(0.160–0.502)
Uninsured, self-pay	1.383	(0.503–3.806)	0.494	(0.165–1.476)
Chronic disease present in household?				
No	1.000		1.000	
Yes	14.223	(6.316–32.030)	7.524	(3.285–17.233)

Table 3 shows the rate of catastrophic health expenditure for households affected by different chronic diseases. Overall, households with members suffering from chronic diseases had a greater chance of experiencing catastrophic health expenditure than households without. In particular, 6.2% of households with members suffering from neoplasm, 12.9% with cerebrovascular disease, 9.6% with diabetes, 7.0% with arthritis, 23.6% with chronic kidney disease, 7.3% with hypertension, and 11.7% with IHD experienced catastrophic health expenditure. Among these households, those with members suffering from chronic kidney disease had the highest catastrophic health expenditure rate.

**Table 3:** **Catastrophic health expenditure and chronic disease**

**Variables**	**Catastrophic health expenditure occurrence by household**		**Catastrophic health expenditure non-occurrence by household**		**Total**	**p-value**
	**Frequency**	**%**	**Frequency**	**%**		
Neoplasm						<.0010
No	18	0.5	3,304	99.5	3,322	
Yes	230	6.2	3,454	93.8	3,684	
Cerebrovascular disease						<.0010
No	212	3.2	6,515	96.8	6,727	
Yes	36	12.9	243	87.1	279	
Ischemic heart disease						0.0021
No	226	3.3	6,592	96.7	6,818	
Yes	22	11.7	166	88.3	188	
Diabetes mellitus						<.0010
No	163	2.7	5,958	97.3	6,121	
Yes	85	9.6	800	90.4	885	
Arthritis						<.0010
No	104	2.1	4,832	97.9	4,936	
Yes	144	7.0	1,926	93.0	2,070	
Chronic kidney disease						0.1137
No	239	3.4	6,729	96.6	6,972	
Yes	9	23.6	29	76.4	34	
Hypertension						<.0010
No	90	1.9	4,753	98.1	4,843	
Yes	158	7.3	2,005	92.7	2,163	

Table 4 shows the results for associations between chronic diseases and catastrophic health expenditure. Households affected by neoplasm were twice as likely to experience catastrophic health expenditure as households that were not affected. The odds of experiencing catastrophic health expenditure were 2.0 times higher for households with cerebrovascular disease, 1.7 times higher for those with diabetes, and 3.2 times higher for those with chronic kidney disease. The latter exhibited the highest odds ratio associated with any type of chronic disease. No statistically significant associations were detected between catastrophic health expenditure and IHD, arthritis, or hypertension.

**Table 4:** **Associations between chronic diseases and catastrophic health expenditure**

**Variables**	**Unadjusted**		**Adjusted** ^**a**^	
	**OR**	**95% CI**	**OR**	**95% CI**
Neoplasm				
No	1.000		1.000	
Yes	8.557	(4.774–15.336)	2.034	(1.049–3.946)
Cerebrovascular disease				
No	1.000		1.000	
Yes	3.098	(2.175–4.413)	1.959	(1.311–2.925)
Ischemic heart disease				
No	1.000		1.000	
Yes	2.953	(1.916–4.550)	1.460	(0.906–2.352)
Diabetes mellitus				
No	1.000		1.000	
Yes	2.586	(2.052–3.260)	1.700	(1.297–2.228)
Arthritis				
No	1.000		1.000	
Yes	1.563	(1.396–1.751)	1.119	(0.963–1.299)
Chronic kidney disease				
No	1.000		1.000	
Yes	3.177	(1.271–7.941)	3.236	(1.116–9.382)
Hypertension				
No	1.000		1.000	
Yes	2.022	(1.690–2.419)	1.143	(0.912–1.432)

^a^Odds ratio obtained using multiple logistic regression models adjusted for head of household’s sex, age, economic level, level of education, and type of job, as well as household composition and type of medical insurance.

Households in the lowest 20% of incomes that did not receive assistance from Korea’s medical aid program were 3.4 times more likely to experience catastrophic health expenditure than households that did receive assistance. Thus, the majority of families in the lowest 20% income bracket were vulnerable to experiencing catastrophic health expenditure. The protective association seen between medical aid and catastrophic health expenditure were similar in the 5%, 10%, and 15% income brackets (Table 5).

**Table 5:** **Association between membership in the Korean medical aid program and catastrophic expenditure occurrence among households in the lowest income quintile** ^**a**^

**Medical aid beneficiary**	**Income bracket**							
	**5%**		**10%**		**15%**		**20%**	
Yes	1.000		1.000		1.000		1.000	
No	6.377	(2.193–18.542)	4.313	(2.111–8.812)	3.522	(1.896–6.542)	3.370	(1.902–5.971)

^a^OR: Odds ratio obtained using multiple logistic regression models adjusted for head of household’s sex, age, level of education, and type of job.

## Discussion and conclusion

In this study, households headed by women and middle-aged individuals (40–59 years) had significantly lower catastrophic health expenditure rates, as did households that benefited from Korea’s medical aid program. The health care system in Korea has two components, health insurance and medical aid. The national health insurance system, which is managed comprehensively in the form of social insurance, is funded by contributions from beneficiaries and provides coverage to all citizens. The other component, Medical Aid, was started in 1977 as a form of public assistance to guarantee the minimum standard of living to low-income households by providing almost free medical services, and is funded by general revenue [22]. Our results showed that households that benefited from Korea’s medical aid program had significantly lower catastrophic health expenditure rates, which is likely due to the fact that medical aid beneficiaries have very low OOP health payments. At least for low-income families, it appears that medical aid contributes more to financial security than alternative insurance programs. In contrast, households of low economic status, those with elderly members, and those containing individuals with chronic diseases had significantly higher odds of experiencing catastrophic health expenditure.

When comparing the demand curve for medical services with the demand curves for other products, it is evident that medical services have low profit elasticity. This means that when there is a need for medical services in a low-income family, low purchasing power does not stop that household from purchasing health care. This leads to high health care expenditures relative to purchasing ability, which can result in low-income households falling deeper into poverty [23]. Our study divided household income in 2008 into three quintiles and determined the present state of catastrophic health expenditure. The results displayed a clear trend of an increasing burden of medical expenses as income approached the poverty line. Families in the lowest of incomes who had not joined Korea’s medical aid program were 3.4 times more likely to experience catastrophic health expenditure than families who had joined.

In Korea, a significant number of low-income persons have not received Medical-Aid eligibility. Although their income level is below 100 percent of the poverty-line, they are excluded when taking into account their net worth or the income property of their support obligor, which exceeds a certain amount [24]. Approximately 10 percent of the total population of South Korea is below 120 percent of poverty level; of these, 3.16 percent are Medical-Aid beneficiaries and the others are in a blind spot of health care [6,7]. Accordingly, the Korean government could further protect their citizens against CHE by alleviating the strict criteria of support obligor. Similarly, elderly households were 2.8 times more likely to experience catastrophic health expenditure than younger households. As the effects of its aging society becomes more pronounced, Korea must continue to establish counter policies while remaining cognizant of the possibility that catastrophic health expenditures will increase with a growing number of independent, elderly households.

In Korean society, chronic diseases place a tremendous burden on not only individual families but on society as a whole [25]. The government is failing to cope with this important problem, however. Korea’s chronic disease management problems arise from various sources, including a distorted health care delivery system, private medical facilities, the lack of an adequate workforce, and lack of awareness of patients. Therefore, the timing of this study appears fortuitous in terms of raising awareness of this problem by demonstrating the relationship between chronic diseases and catastrophic health expenditures.

Furthermore, looking at the varying effects of different types of chronic diseases provides an important perspective on how financial security can be enhanced according to chronic disease status. In this study, we showed that families with chronic disease were 7.5 times more likely to experience catastrophic health expenditure than families without; households affected by chronic kidney disease were 3.2 times more likely to experience catastrophic health expenditure than other households. The total direct and indirect costs associated with chronic kidney disease were estimated to amount to over 5 trillion KRWs (5 billion dollars) in 2011. Compared with other major chronic diseases, including chronic obstructive pulmonary disease (800 million dollars) and chronic liver disease (3 billion 800 million dollars), the cost of chronic kidney disease are substantial. Although the socioeconomic burden of chronic kidney disease is alarming in Korea, financial protection systems to help patients who have chronic kidney disease have not been developed [23]. In 2013, nonetheless, the Korean government presented a plan for strengthening benefits for four severe diseases: cancer, cardiovascular disease, cerebrovascular disease, and rare and incurable disease. This policy will be implemented by 2016 and all treatments for these diseases are to be covered by health insurance without non-insured medical cost. We suggest that chronic kidney disease warrants consideration for being added to this list, as the odds of experiencing catastrophic health expenditure were 3.2 times higher for those with chronic kidney disease, compared to 2.0 times higher for households with cancer and cerebrovascular disease, in this study. Nevertheless, reference to studies like this research would help in guiding decisions on expanding benefits for particular diseases.

In this study, the rate of catastrophic health expenditure in 2008 was 3.5%. This is similar to the figure of 3.0% that the WHO [14] reported for 2007 using Korea National Statistic’s data. Despite the enhanced security provided by Korea’s medical aid program in the time period studied, the rate of catastrophic health expenditure actually increased from 2007 to 2008, and further research is needed to determine the cause.

Our research differs from previous studies on catastrophic health expenditure in several ways [19,26-29]: First, previous research on catastrophic health expenditure in Korea primarily focused on household budget surveys. Until now, household budget surveys were the only source of information regarding medical costs, which made carrying out systematic studies problematic. For example, household budget survey data do not include information on the diseases from which patients suffered, resulting in difficulties in determining the causes of medical spending. For this reason, the Korea Health Panel provides important data with which to study the catastrophic health expenditure of households. Major diseases affecting household members were recorded in detail, and a yearly record of spending was obtained, another advantage compared to the monthly data provided by household budget surveys. By using Health Panel data, in our study, we could investigate the effect of the type of chronic disease for the first time. As a result, we could determine, using straightforward statistics, the influence that chronic diseases had on household catastrophic health expenditure. This information may have policy implications in terms of elucidating how financial security can be enhanced according to the diseases present in a household. A second advantage of this study is that we employed a method of calculation very similar to that introduced by Xu et al. [10] in their analysis of Korea Health Panel data. Due to the fact that food expenses could not be calculated in a few studies, some research defined catastrophic health expenditure by comparing medical expenditures to a household’s total income [21,30-32]. However, we were able to utilize Korea National Statistics data to calculate a household’s average food expenses in this study. We estimated Korea’s catastrophic health expenditure rate by utilizing proportionate food expenses applied to two sources of data, total income and medical expenditures. Third, unlike previous data analysis that was conducted solely at the household level, we were able to consider the characteristics of individual family members. For example, we could take into account not only whether household members suffered from chronic diseases but also the socioeconomic factors of the head of household.

Nevertheless, there were some limitations associated with our study and with using the Korea Health Panel data. First, this study was cross-sectional in design; thus, possible inverse causality between chronic diseases and catastrophic expenditure are not reflected. Second, the Health Panel relies on data collection from a sample of the population. Therefore, in order to accurately measure the rate of catastrophic medical expenditure for the population as a whole, weighted values must be applied. However, in our research it was not possible to use weighted values. Third, the Korea Health Panel data for 2008 were collected in biannual segments; however, the medical expenditures reflected in the data do not fall exactly within 2008. We combined the two periods of medical expenses, making the assumption that the sum reflected the medical costs incurred in 2008 fairly accurately; nevertheless, there is the possibility that we underestimated catastrophic medical expenditures. Fourth, the health care fees and income reported in the 2008 Korea Health Panel data were drawn from different time periods. Reported income was recorded from 2007, while medical costs were surveyed in 2008. Accordingly, we assumed that the previous year’s income represented the household purchasing power for the next year. Fifth, we collected no information on coping mechanisms (selling assets, bonds, and borrowing from relatives or banks/market etc.) among individuals/households who could not meet out-of-payment health expenditures.

In conclusion, this study examined the present state of catastrophic health expenditure and the factors associated with it in South Korea. We found that despite the fact that health care security has been continuously strengthened, the rate of catastrophic health expenditure in Korea is still high and that this phenomenon is more pronounced in low-income households. We also discovered the existence of blind spots in financial security, such as in those with chronic kidney disease. These results reveal that the current system is not providing a minimal medical safety net for individuals or households. Therefore, greater financial protection of families is needed, especially those from the poorer sections of society [33]. We hope that policies will be created in the future to alleviate such problems.
